# The Role of Epigenetic Modifier Mutations in Peripheral T-Cell Lymphomas

**DOI:** 10.3390/cimb45110563

**Published:** 2023-11-10

**Authors:** Adrian-Bogdan Tigu, Anamaria Bancos

**Affiliations:** 1 Medfuture Research Center for Advanced Medicine, Iuliu Hatieganu University of Medicine and Pharmacy, 400337 Cluj-Napoca, Romania; ani_maribancos@yahoo.com; 2 Academy of Romanian Scientists, Ilfov 3, 050044 Bucharest, Romania; 3 Department of Hematology, Iuliu Hatieganu University of Medicine and Pharmacy, 400012 Cluj-Napoca, Romania

**Keywords:** PTCL, epigenetic modifiers, therapy, mutations

## Abstract

Peripheral T-cell lymphomas (PTCLs) are a group of diseases with a low incidence, high degree of heterogeneity, and a dismal prognosis in most cases. Because of the low incidence of these diseases, there have been few therapeutic novelties developed over time. Nevertheless, this fact is changing presently as epigenetic modifiers have been shown to be recurrently mutated in some types of PTCLs, especially in the cases of PTCLs not otherwise specified (PTCL-NOS), T follicular helper (TFH), and angioimmunoblastic T-cell lymphoma (AITL). These have brought about more insight into PTCL biology, especially in the case of PTCLs arising from TFH lymphocytes. From a biological perspective, it has been observed that ten-eleven translocators (TET2) mutated T lymphocytes tend to polarize to TFH, while Tregs lose their inhibitory properties. IDH2 R172 was shown to have inhibitory effects on TET2, mimicking the effects of TET2 mutations, as well as having effects on histone methylation. DNA methyltransferase 3A (DNMT3A) loss-of-function, although it was shown to have opposite effects to TET2 from an inflammatory perspective, was also shown to increase the number of T lymphocyte progenitors. Aside from bringing about more knowledge of PTCL biology, these mutations were shown to increase the sensitivity of PTCLs to certain epigenetic therapies, like hypomethylating agents (HMAs) and histone deacetylase inhibitors (HDACis). Thus, to answer the question from the title of this review: We found the Achilles heel, but only for one of the Achilles.

## 1. Introduction

Peripheral T-cell lymphomas (PTCLs) are a group of diseases in which the malignant clone starts from a T lymphocyte that underwent T-cell receptor (TCR) rearrangement. Currently, there are 29 entities of PTCL, as described in 2016 by the World Health Organization (WHO), with most of them having specific clinical presentation and therapeutic management, which in turn reflects the heterogeneity of these diseases [1,2]. This, with the addition of low incidence, leads to difficulty in researching these diseases and conducting significant clinical trials on them. Nevertheless, there has started to be an increase in the advancements made for PTCLs, with a particular focus on the recurrent epigenetic changes that occur, especially in the case of entities in which the malignant clone started from a T follicular helper (TFH) lymphocyte. These are represented mostly by angioimmunoblastic T-cell lymphoma (AITL) and PTCL not otherwise specified (NOS) TFH variants. PTCL-NOS has a high heterogenicity, with considerable differences from case to case in terms of their clinical, pathological, and genetic characteristics, making diagnosis and treatment very challenging. PTCL-NOS originates from mature T-cells, and the clinical aspects involve enlarged lymph nodes, fever, fatigue, night sweats, weight loss, and sometimes skin rashes. PTCL-NOS includes abnormal T-cell infiltrations in lymph nodes and proximal tissues, with features that vary from case to case [3,4].

AITL accounts for 30% of T/NK cell lymphomas, frequently diagnosed in middle-aged and elderly patients. Clinical manifestations include generalized lymphadenopathy and extranodal sites like the liver, spleen, or bone marrow. Moreover, laboratory evaluation usually highlights elevated rheumatoid factors, positive anti-smooth muscle antibodies, and other immune abnormalities; thus, patients show poor outcomes and less than three years median survival [5,6,7]. AITLs have recurrent mutations in genes encoding TET2, IDH2 R172, and DNMT3A, with some mutations being identified in non-tumor cells of patients with AITL and even in some healthy donors; however, IDH2R172 and RHOAG17V mutations are confirmed to tumor cells; all these may help in targeted therapy and personalized therapeutical approaches [8,9,10,11,12].

PTCL-TFH includes cases of TFH lymphomas that do not have sufficient pathological features to be included as AITLs; thus, some AITL features are common for PTCL-TFH, like the diffuse proliferation of neoplastic cells, but without a pronounced inflammatory background. The subtype is characterized by TET2, DNMT3A, and RHOAG17V mutations, with a higher frequency of TET2, though without IDH2R172 mutations [3,13]. PTCLs represent one out of ten non-Hodgkin lymphomas (NHLs) and a large proportion of aggressive lymphomas. The most common subtype of PTCLs is represented by NOS in North America and Europe, with a frequency of around 30%, while in Asia, NOS represents the second most common subtype with 22% frequency [4,14]. 

PTCL-NOS risk factors include a family history of psoriasis, celiac disease, or other hematological malignancies; moreover, other factors, such as long-term smoking, are risk factors for all PTCLs [15,16]. It was observed that PTCL-NOS is frequently diagnosed more than five years post-transplant with extranodal sites [17]. Furthermore, in Asia, EBV-associated PTCL-NOS is very common and is associated with poor outcomes [18]. 

Because of the recurrent epigenetic changes in PTCL, there have been several clinical trials testing hypomethylating agents (HMAs) or histone deacetylase inhibitors (HDACis) in PTCLs, with impressive results being obtained [19,20].

Epigenetic alterations play an important role in the pathogenesis and development of peripheral T-cell lymphoma due to the dysregulation of gene expression and cell signaling [21]. At this moment, the first-line therapy for PTCLs is Cyclophosphamide+ doxorubicin+ vincristine + prednisolone (named CHOP) or other CHOP-like regimens; however, most PTCLs are very aggressive and have poor clinical outcomes [22,23,24]. In recent years, PTCLs have gained attention, and great advancements have been made in understanding the pathology and developing novel therapies; thus, CHP with brentuximab vedotin was approved as a frontline treatment [25].

Abnormal activity of the histone deacetylases (HDACs) affects gene expression and can lead to the silencing of specific tumor suppressor genes or can activate specific oncogenes. Several mutations in epigenetic modifying genes such as TET2, IDH2-R172, RHOA, IDH2, or DNMT3A have been reported in PTCL cases, with DNMT3A, IDH2, and TET2 mutations being the most frequent mutations identified in AITL and PTCL-NOS, and these are associated with disease progression [10,26,27]. 

**Figure 1:**
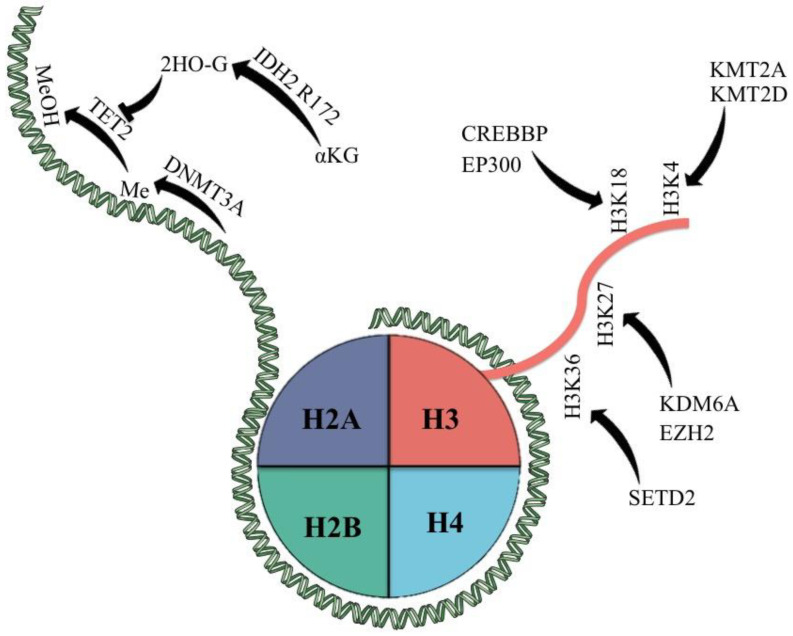
Epigenetic modifiers most commonly mutated in PTCL and their classic targets. αKG = α-keto glutarate; 2HO-G = 2-hydroxy glutarate; Me = methyl group; MeOH = hydroxy methylated group.

Because of these, we will further discuss the current knowledge of mutations affecting epigenetic modifiers in PTCLs (Figure 1) and their current impact on therapeutic management. 

Epigenetic modifications are key for the development and progression of PTCL; DNA methylation and histone modifications can lead to dysregulation in gene expression in PTCL, where aberrant DNA methylation or histone acetylation can activate oncogenes, promoting PTCL progression. Any epigenetic modification can serve as prognostic markers in PTCL and is not limited to this particular disease. Patterns in DNA methylation, demethylation, and histone markers can be predictors of treatment response, and patient overall survival or can be used to guide physicians to choose better therapeutic alternatives. Current therapies target epigenetic regulators, and many of the epigenetic regulators were evaluated in clinical trials to design and approve drugs that can restore normal epigenetic patterns and inhibit tumor cell growth. Studies in this field reveal the fundamental biology of PTCLs and may pave the way for better-targeted therapies for aggressive cancers like PTCLs.

## 2. The Landscape of DNA Methylation and Epigenetic Modifiers

Normal (benign) DNA and cancer DNA have unique and different patterns of methylation; tumor DNA has a lower percentage of methylated cytosine compared to normal DNA [28]. The first described epigenetic modification in cancer cells was reported in 1983 when the Ehrlichs group demonstrated that the 5-methyl cytosine is replaced by unmethylated cytosine [29]. Global hypomethylation of the genomic DNA was observed in several types of cancers [30,31,32,33]; however, the CpG islands that are overlapping the promoters were hypermethylated. The regional hypermethylation may occur in early tumorigenesis and can be associated with tumor progression, serving as an indication for survival. Moreover, the global hypomethylation of the DNA can be associated with tumor progression [34,35,36].

These differences in the grade of methylation between normal and tumor DNA are exploited by researchers worldwide to determine if there are some methods to discriminate between methylated and non-methylated DNA and to predict cancer evolution by analyzing extracted DNA from both solid and liquid biopsy [37]. Thus, epigenetic modification in the DNA and the methylation patterns can be used to predict tumor progression and survival by evaluating the methylators and the DNA methylation grade. 

**Figure 2:**
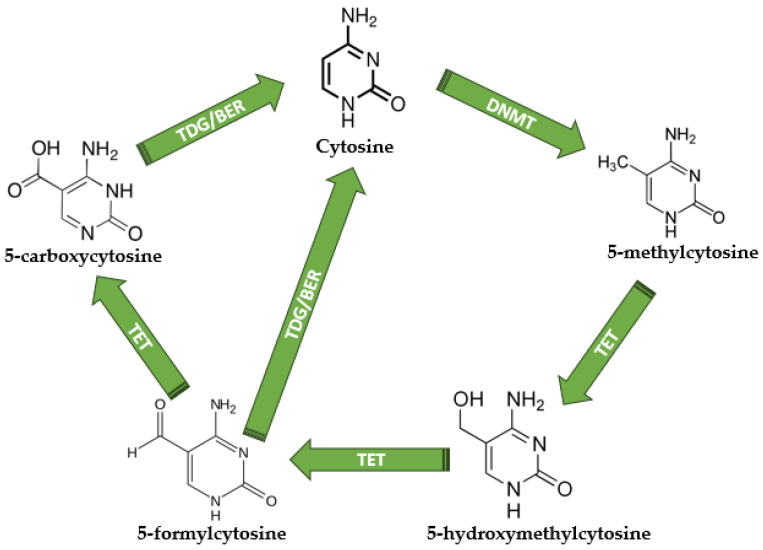
The DNA methylation and demethylation mechanisms. Unmethylated cytosine becomes 5-methylcytosine under DNMT activity; therefore, TETs are responsible for 5-hydroxymethylcytosine, 5-formylcytosine, and 5-carboxycytosine production. TDG is responsible for the direct conversion of 5-formylcytosine and 5-carboxycytosine into normal unmethylated cytosine, terminating the demethylation cycle. DNMT—DNA methyltransferases; TET—Ten-eleven translocations; TDG—thymine DNA glycosylase; BER—base excision repair. All chemical structures were designed using MedChem Designer software version 5.5.

The biological processes of DNA methylation and demethylation have key functions in gene regulation and have a crucial impact on the development of cells [38]. The methylation/demethylation usually occurs in CpG islands, and the process is modulated by de novo DNA methyltransferases (DNMTs), which add the methyl group to the cytosine, and ten-eleven translocators (TET), which are converting 5-mC (5-methylcytosine) into 5-hmC (5-hydroxymethylcitosine), 5-fC (5-formylcytosine), and 5-caC (5-carboxycytosine) [39,40]. During the demethylation cascade, both 5-caC and 5-fC can be converted into normal unmethylated cytosine, a process catalyzed by the thymine DNA glycosylase (TDG), which allows base excision repair (BER) and the generation of unmethylated cytosine [40]. Since the characterization of DNA structure in 1953 [41], it is well known that DNA contains four nitrogenous bases: adenine (A), thymine (T), guanine (G), and cytosine (C), while the methylated cytosine is considered the “fifth base” of DNA [42]. The methylation/demethylation process and its key players are depicted in Figure 2.

DNA methylation has a key role in cell biology; the process regulates gene expression and is involved in monoallelic silencing and centromere stability [43]. It is one of the most abundant and studied epigenetic modifications, and the methylation patterns seem to be different in normal cells compared to tumor cells. In cancer, DNA global hypomethylation events occur in key regions such as enhancers and promoters of critical genes, which lead to overexpression of some oncogenes or downregulation of the expression of regulatory genes [43].

The human genome contains around 28 million CpG sites, of which more than two thirds are methylated in normal somatic cells. The CpG sites are not evenly distributed, as the bulk of the genome contains fewer CpG sites, while the rest are clustered in CpG islands. The CpG islands are 500–1000 base pairs long, and usually, they are located in the promoter regions of the genes [44]. The unmethylated CpG sites are a binding platform for the transcription factors that control gene activity; thus, the methylation of the CpG sites near a gene can silence the gene. DNA methylation levels in the enhancer’s region are correlated with gene expression activity; thus, lower levels of methylation are associated with increased transcriptional activity [45].

However, during tumorigenesis, normal epigenetic processes are disrupted. Thus, DNA methylation patterns are modified. This aspect is characterized by global hypomethylation with specific regional hypermethylation of the promoter’s CpG islands, commonly associated with the silencing of specific tumor suppressor genes or genes that control cell growth and proliferation [46]. Global hypomethylation in cancer reduces the genomic stability of the cells as reduced DNMT1 levels have been indicated to facilitate a favorable environment for higher mutation rates and tumor development. Low DNA methylation is also related to aberrant expression of oncogenes and transposable elements, resulting in the deregulation of critical cellular processes that control cell growth, differentiation, and proliferation [47,48].

DNMT3A and 3B are adding the methyl group to normal cytosine and generating 5-mC, while DNMT1 is propagating the modification following replication. In contrast with the methylation process, other proteins are oxidizing the 5-mC and promoting demethylation [49,50]. To maintain the promoter CpG islands unmethylated and to keep the globally hypomethylated DNA in normal cells, the DNA demethylation mechanism is crucial. The 5-hmC is the result of the first demethylation step, given the activity of TET proteins. As DNMTs add methyl to the normal cytosine, TET proteins are responsible for demethylation by oxidating 5-mC to 5-hmC [51]. Several studies evaluated the loss of TET proteins, and the results highlighted an increased hypermethylation of the enhancers and promoters, resulting in impaired cell development [52,53,54].

Oxidized methylcytosines and methylated cytosines are crucial in the maintenance of the identity of each cell, and these modifications are part of the gene expression biological processes that do not alter the DNA sequence. 5-mC patterns contribute to the cellular identity and functions, silencing tissue-specific genes that should be inactive in other tissues, while 5-hmC and other oxidized cytosines contribute to the gene expression dance and DNA demethylation steps [55,56,57].

However, there are situations when DNA methylators such as DNMTs and TET proteins suffer mutations, and the downstream-regulated pathways are significantly altered, with reverberations in cell differentiation and developmental processes. Mutations in DNMTs can induce abnormal DNA methylation patterns, silencing inappropriate genes or activating unneeded genes that, in normal conditions, are silenced [58,59,60]. On the other hand, mutations occurring in TET impact the demethylation process, and the results are translated into an abnormal DNA methylation pattern commonly associated with specific disorders, including leukemia and other types of cancer. Somatic mutations of the DNMT3A gene were correlated with lower overall survival in patients with acute myeloid leukemia, and dysregulation of the DNMTs in leukemia can contribute to disease progression by silencing tumor suppressor genes [55,61]. Three TET proteins are involved in 5-mC oxidation; thus, alterations in TET were found in various myeloid malignancies and are related to an unfavorable prognosis [62].

In 2016, Lemonnier and his group highlighted that treatment with azacytidine can sustain response in patients with AITL [63]. Treatment with the hypomethylating agents’ showed efficacy in myelodysplastic syndrome and acute myeloid leukemia, a response that appears to correlate with IDH1/2, TET2, and DNMT3A mutations [64,65,66]. These findings suggest that hypomethylating agents could act as an efficient therapeutic alternative in PTCL-TFH. It was reported that patients suffering from AITL and chronic myelomonocytic leukemia had a good outcome and reached remission after treatment with azacytidine [67,68]. The study presented by Lemonnier included 12 patients with AITL who received azacytidine, 9 with a positive response, 6 with a complete response, and 3 with a partial response, with an overall response rate of 75%. In 12 out of 12 patients, TET2 mutations were detected; 58% had 2 mutations, 33% had DNMT3A mutations, and 41% had RHOA mutations, while only one had the IDH2 R172 mutation [63].

Mutations in DNMTs and TET genes, abnormalities in IDH2, and changes in histone markers can change the epigenetic landscape and lead to abnormalities in cellular development, which are also responsible for cancer development.

### 2.1. The Role of Ten-Eleven Translocations (TET)

Ten-eleven translocations are a group of enzymes represented by TET1, TET2, and TET3, which are responsible for methylcytosine oxidation. TET acts by oxidizing 5-methylated cytosines, generally from a CpG island to 5-hydroxymethyl cytosine, and then plays a role in the next oxidation steps of the hydroxymethyl group to formyl and carboxyl, the latter step leading to a return to the demethylated cytosine conversion facilitated by the TGD/BER complex. Nevertheless, there are also studies discussing the role of 5-hydroxymethyl cytosine as more than an intermediate in the demethylation process, representing a proper epigenetic mark by itself [69,70].

Because of its physiologic activity, TET inhibition was correctly inferred to generate global hypermethylation. This has been studied in acute myeloid leukemia (AML) in the case of TET2 mutations, which have been shown to be generally represented by loss-of-function mutations [62].

In mouse models, TET2 loss-of-function mutations have been shown to increase the hematopoietic stem cell (HSC) pool, leading to an increased probability of malignant transformation in both myeloid and lymphoid diseases. Myeloid diseases generated by this model are represented by the most common entities, like AML, chronic myelomonocytic leukemia (CMML), and myeloproliferative neoplasms (MPNs) [71].

The B-lineage lymphoid disease that occurs more frequently in this model is represented by diffuse large B-cell lymphoma; others show that TET2 can represent the first hit leading to germinal center hyperplasia, and BCL2 overexpression represents the second event in this model that leads to the formation of DLBCL [72].

The T-lineage lymphoid diseases that occur more frequently in TET2 mutant mice are PTCLs, in which the cell of origin is represented by the TFH lymphocyte. More specifically, AITL and PTCL-NOS TFH represent these diseases [73,74,75]. This was also confirmed in the clinical scenario, as it has been shown that AITL and PTCL-NOS TFH are present in a quarter to half of the cases of mutations in TET2 [10,76,77]. Clinically, it is not generally considered that TET2 mutations influence the prognosis of PTCL. Nonetheless, it has been shown that TET2 mutations correlate with high-risk prognostic factors like the International Prognosis Index (IPI) and a platelet count under 150,000/µL [76]. Aside from PTCL that occurred from a TFH lymphocyte, there have been studies showing that TET2 mutations can also be present in adult T-leukemia/lymphoma (ATLL) [78] and in enteropathy-associated T-lymphoma [76], although in a lower percent of cases.

One question that arises is: “Why are TET2 mutations associated with a TFH phenotype?”. The answer to this can be at least partially inferred from the role of TET2 in T-cell polarization. More specifically, it has been shown that TET2 mutations repress the expression of FOXP3 in Tregs, leading to a suppression of these cells and stimulation of effector T-cells [79]. Moreover, the same article has shown that CD4+/FOXP3− cells presenting a mutant TET2 have a higher probability of developing a TFH phenotype, a fact that leads to the consideration that this might be one of the mechanisms that increases the likelihood of developing AITL or PTCL-NOS TFH [79]. Others have also shown that TET2 mutations lead to FOXP3 destabilization in Tregs and an increase in IL17 secretion [80]. Nonetheless, it must be mentioned that conflicting studies show that TET2 deletion leads to an inhibition of Th1 and Th17 polarization and cytokines associated with these states [81].

**Figure 3:**
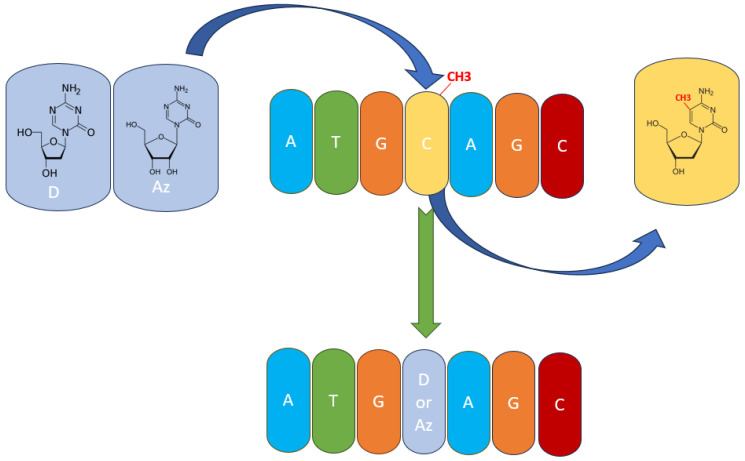
The mechanism of action of hypomethylating agents. Azacytidine (Az) and decitabine (D) are incorporated into the DNA polynucleotide chain and replace 5-mC.

Therapeutically, PTCL subtypes presenting TET2 mutations or TET2 inhibition from other causes could be thought of as having a sensitivity to hypomethylating agents (HMAs) like azacytidine (AZA) and decitabine (DAC). This has also been shown in clinical trials, with AZA presenting high efficacy in PTCL and, more specifically, in AITL [1,20,67]. Both AZA and DAC have chemical structures like cytosine, with the main difference being that their aromatic ring has one additional nitrogen, which replaces the carbon atom in the 5′ position where the methylation takes place (Figure 3). Thus, DNMT3A and DNMT3B are unable to add the methyl group at the 5′ position, because the nitrogen atom has no more free electrons to share [82,83,84]. Both AZA and DAC have the potential to treat peripheral T-cell lymphomas because of their ability to target abnormal DNA methylation patterns. Some subtypes of PTCLs respond better to hypomethylating agents than other subtypes; however, the outcome has mixed results in clinical trials; some patients respond well to the therapy, while others suffer because of the side effects. Thus, the safety and tolerability of these two hypomethylating agents are important considerations when choosing hypomethylation agent therapies in PTCLs. Studies are ongoing to optimize the use of hypomethylating agents in PTCL and to identify possible biomarkers and combination strategies that can enhance the efficacy of the therapy.

### 2.2. The Role of IDH2

Physiologically, IDH1/2 converts isocytric acid to α-ketoglutarate. It has been shown in AML that IDH1 R132 and IDH2 R140 or R172 lead to the conversion of α-keto-glutarate to 2-hydroxy-glutarate, which leads to the inhibition of TET2 with similar effects on the methylation and transcription profiles [85]. These mutations can lead to abnormalities in metabolic processes and may contribute to the progression and development of PTCL and other types of cancer. Mutations in IDH2 can induce abnormal levels of 2-hydroxyglutarate, a metabolite that can disrupt normal cellular metabolism; moreover, the metabolite is responsible for DNA and histone demethylase inhibition, leading to aberrant methylation patterns and modulating gene expression [86,87]. This equivalence between the two mutations in AML is also seen because of the mutual exclusivity presented between these mutations [85]. Conversely, in PTCL, the most common IDH1/2 alteration is represented by IDH2 R172, with quite rare occurrences of the other pathogenic mutations in IDH1/2. This mutation has been most commonly observed in AITL [88] and, interestingly, does not present mutual exclusivity with TET2 mutations, showing that it might have other or additional roles in AITL compared to AML [10,88,89]. Moreover, there was no association between IDH2 R172 and clinical or pathologic features, which in the case of TET2 mutations were observed [76], adding another argument for the differential impact of IDH2 R172 between AML and AITL. Also, it has to be considered that 2-hydroxy-glutarate has the potential to inhibit other enzymes, like the Jumonju C-Domain histone demethylase family [90,91], or through inhibition of PHD and upregulation of HIF1α [92]. Experimental evidence on CD4+ T-cells and AITL patients shows that IDH2 R172 mutations not only lead to an increase in 2-hydroxy-glutarate and hypermethylation of certain promoters but also an increase in H3K27me3 marks [90].

From a therapeutic perspective, following the model of AML, IDH2 R172 could represent a potential target for IDH2 inhibitors, like Enasidenib, or could represent potential targets for HMAs [93]. Nonetheless, these suppositions must be validated through clinical trials.

### 2.3. DNMT3A

DNMT3A is a de novo methyl transferase acting as a homotetramer on CpG islands, generating a hemimethylated cytosine, followed by complete methylation by DNMT1 [94]. DNMT3A mutations are common in hematological malignancies such as acute myeloid leukemia and myelodysplastic syndromes. Mutations in DNMT3A lead to overstimulation of DNA methylation, altering the expression of genes involved in cell differentiation or genes that regulate hematopoiesis. The impaired differentiation of the hematopoietic stem cells can lead to the accumulation of immature cells and interfere with normal blood cell production [94,95]. The most common variant mutation of DNMT3A, both in AML and PTCL, is represented by R882, which has been shown to inhibit homotetramer formation, leading to an 80% reduction in DNMT3A activity and a generalized hypomethylation [96]. In AML, DNMT3A mutations were observed to occur at frequencies between 20 and 30% depending on the cytogenetic risk group, while in PTCL, it was observed that DNMT3A mutations occur more frequently in AITL compared to other subtypes of PTCL, at approximately 30% of the cases with a significant overlap with TET2 mutations, while in PTCL-NOS or PTCL-NOS TFH, they occur between 4 and 10% [3,10,11,89]. There are also authors hypothesizing that DNMT3A/TET2 mutations represent a first hit, which is followed by a second hit in RHOA/IDH2 on the path for the pathogenesis of AITL [97]. Although, as mentioned before, TET2 mutations lead to a skewed generation of TFH cells, this is not the case for DNMT3A mutations. It has been observed that DNMT3A inhibition leads to the upregulation of FOXP3, leading to an anti-inflammatory effect [98]. Nonetheless, it has been observed in a mouse model that DNMT3A deletion results in an accumulation of T-cell progenitors in the thymus and lower apoptosis rates of these progenitors, with an increased risk of developing T-acute lymphoblastic leukemia (T-ALL) [99]. Others have shown that DNMT3A+/− mice have an increased risk of developing CD8+ lymphoma, with the second event in these cases being modeled for p53 downregulation [100]. The study conducted by Herek et al. highlighted that DNMT3A mutations define a cytotoxic subset in PTCL-TBX21 with prognostic value, clarifying the heterogenicity of PTCL-NOS [101].

### 2.4. Histone Marks

Histone marks have been known now for more than half a century, with more information about these posttranslational changes being published every year. These marks have various effects on gene transcription and chromatin accessibility, with several enzymes implicated in managing these marks. They are broadly classified as writers (insert a mark), readers (recognize a mark), and erasers (erase a mark) [102,103]. Mutations in histone markers significantly alter gene regulation and disrupt epigenetic regulation. Mutations in histone H3 change the amino acid residues, which are targets for methylation and other modifications; thus, normal gene expression is disturbed [104,105]. Moreover, there are histone modification enzymes that can undergo modifications, leading to dysregulated gene expression patterns that can lead to cancer or other diseases [106,107]. Histone methyltransferases can suffer mutations resulting in abnormal histone methylation patterns, or histone acetyltransferases can gain mutations that lead to gene silencing, processes that are responsible for cancer development [108,109]. Taken together, all histone mark mutations can dysregulate the epigenetic landscape and contribute to various diseases. Mutations in these enzymes were observed to occur frequently in PTCL, more specifically in AITL and PTCL-NOS TFH [110]. The genes most frequently observed to be altered in PTCL are represented by KMT2D (H3K4 methyltransferase), KMT2A (H3K4 methyltransferase), SETD2 (H3K36 methyltransferase), KDM6A (H3K27 demethylase), CREBBP (H3K18 acetyltransferase), EP300 (H3K18 acetyltransferase), and EZH2 (H3K27me3 reader) [111,112,113,114]. Wu et al. [115] described that gene fusion involving tumor protein 63 (TP63), which is correlated with poor survival in T and B-cell lymphomas, is involved in tumor survival through EZH2. The research group highlighted that mice expressing TBL1XR1:TP63, which is the most common TP63 fusion, develop T-cell lymphomas and the fusion coordinates the recruitment of nuclear receptor corepressor (NCoR)-histone deacetylase (HDAC3) and lysine methyltransferase 2D (KMT2D), which are necessary for fusion-dependent survival. Furthermore, it upregulates MYC and polycomb repressor complex 2 (PRC2), which are components of EZH2. Furthermore, one patient with TP63 rearrangement in lymphoma showed a good response to valemetostat (EZH2 inhibitor), indicating that some fusions can increase therapeutic vulnerability to EZH2 inhibitors. The distribution of chromatin states for both EZH1 and EZH2 play a significant role in the global regulation of histone methylation in aggressive lymphomas; thus, it is essential to inhibit them to achieve a good outcome in EZH2 overexpression or EZH2 mutations in histone-modifying genes as well as in precancerous cells that are affected by epigenetic disruptions [116].

From a survival perspective, patients harboring these mutations were shown to have worse overall survival [111]. Nonetheless, these patients might also be the ones to benefit the most from therapy with HDACi, which is approved as monotherapy in PTCL and, additionally, represents a good basis for forming doubled therapies for PTCL that have already shown encouraging results [1]. There have been studies showing that these mutations, although increasing the mortality risk, also increase the chance of a patient responding to chidamide in the case of PTCL-NOS [111]. Others have shown that EZH2 mutations can be found in ATLL and increase the chance of patients responding to epigenetic therapy [117]. Also, considering the previous chapter of the current review, an important therapeutic strategy is represented by the combination of an HMA and an HDACi, which has been shown to increase the interaction between KMT2D and PU.1, leading to an inhibition of pERK, which is known to be upregulated in PTCL [118,119]. Moreover, this hypothesis was confirmed in a phase 1 clinical trial, which has shown that azacytidine and romidepsin have marked efficacy in PTCL [20].

## 3. Conclusions

Considering the latest advancements in the field of PTCL, it can be said that subtypes of PTCL arising from a TFH lymphocyte can present sensitivity for therapies encompassing an HMA and an HDACi. Nevertheless, it must be mentioned that the mutations discussed in this review are not present as frequently in all subtypes of PTCL, in which case novel approaches must be developed. Finally, to answer the question from the title of this review: “We found the Achilles heel, but only for one of the Achilles”.

## 4. Further Directions

The characterization of specific mutations in epigenetic modifiers, such as DNMT3A, IDH2, and TET, in histone markers and in different subtypes of PTCL is essential to understanding how these mutations affect the epigenetic landscape and gene expression. Potential biomarkers associated with epigenetic modifier mutations are important for a better prediction of the response to therapies, while a deep understanding of the functional consequences of epigenetic modifier mutations on signaling pathways, tumor microenvironment, and gene expression may lead to the development of targeted therapies designed for each abnormal modification. Finally, combinatorial therapies that target specific pathways or immune checkpoints need to be explored in a tight collaboration between clinicians, researchers, and pharmaceutical companies to provide therapeutic alternatives in PTCLs.

## Data Availability

Not applicable.
